# Twelve-Month Outcomes of Standalone iStent Infinite in Primary Open-Angle Glaucoma

**DOI:** 10.3390/jcm15114215

**Published:** 2026-05-29

**Authors:** Arkadiy Yadgarov, Dana M. Hornbeak, Deana Davidova

**Affiliations:** 1 OMNI Eye Services, Atlanta, GA 30342, USA; yadgarovmd@gmail.com (A.Y.); deanadavidova@gmail.com (D.D.); 2 Glaukos Corporation, 1 Glaukos Way, Aliso Viejo, CA 92656, USA

**Keywords:** iStent infinite, microinvasive glaucoma surgery, trabecular micro-bypass, intraocular pressure, standalone, glaucoma

## Abstract

**Background/Objectives**: Evaluation of real-world outcomes of standalone implantation of the third-generation trabecular micro-bypass device (iStent infinite) in eyes with mild to severe primary open-angle glaucoma (POAG). **Materials**: This retrospective, uncontrolled consecutive case series included eyes undergoing standalone iStent infinite implantation at a single U.S. practice. Outcomes were assessed through 12 months, including changes in intraocular pressure (IOP) and medication burden (primary), proportions achieving IOP ≤ 12/15/18 mmHg and medication categories (secondary), and safety. Subgroup analyses were completed based on preoperative IOP, glaucoma severity, and medication burden. **Results**: Fifty-one eyes (mean age 66.5 ± 10.8 years) were included. Mean baseline IOP was 20.1 ± 5.4 mmHg on 2.1 ± 1.2 medications. At month 12, the mean IOP decreased to 16.0 ± 3.6 mmHg (−4.1 mmHg, −20.4%; *p* < 0.001), and mean medications decreased to 1.5 ± 1.2 (−28.6%; *p* < 0.001). The proportion of eyes achieving IOP ≤ 18/15/12 mmHg increased from 41.2%/15.7%/3.9% to 79.6%/42.9%/16.3%, respectively (all *p* < 0.001). Medication-free eyes increased from 15.7% to 30.6%, while eyes requiring ≥ 3 medications decreased from 47.1% to 20.4%. Eyes with baseline IOP > 18 mmHg achieved greater IOP reduction (−27.8%), whereas eyes with baseline IOP ≤ 18 mmHg maintained stable IOP with reduced medications. Kaplan–Meier analysis demonstrated 12-month freedom from incisional reintervention of 92.2%. No intraoperative complications occurred. Transient self-resolving hyphema was observed in 3.9% of eyes. A secondary incisional surgery was performed in four eyes (7.8%); no vision-threatening complications were reported. **Conclusions**: Standalone iStent infinite implantation resulted in significant IOP and medication reductions with a favorable safety profile over 12 months, with outcomes aligned with preoperative treatment goals. These results suggest potential benefit as a less invasive real-world glaucoma intervention, warranting confirmation in larger prospective studies.

## 1. Introduction

Glaucoma is a leading cause of irreversible blindness worldwide, with primary open-angle glaucoma (POAG) representing the most common subtype [1]. Lowering intraocular pressure (IOP) remains the only proven strategy to slow glaucomatous progression. Traditionally, treatment has followed a stepwise paradigm in which topical medications are used as first-line therapy, with filtration surgery reserved for advanced disease. However, topical medications are associated with poor adherence, ocular surface disease, local and systemic side effects, and IOP fluctuations that may contribute to disease progression [2,3,4,5]. Conversely, traditional filtration procedures carry substantial risks, including hypotony, bleb-related complications, and endophthalmitis, which often limit their use to advanced disease [6,7,8].

These limitations have driven a shift toward earlier procedural intervention—the interventional glaucoma paradigm—encompassing selective laser trabeculoplasty (SLT), minimally invasive glaucoma surgery (MIGS), and sustained-release pharmacologic implants [9,10,11,12,13,14,15,16]. Within this framework, trabecular micro-bypass stents represent one of the most widely studied MIGS technologies. The iStent infinite system—a third-generation device consisting of three preloaded titanium stents designed to facilitate multi-quadrant aqueous outflow through Schlemm’s canal—has demonstrated significant IOP reduction in the FDA registration trial and superior outcomes compared with the Hydrus Microstent in the randomized INTEGRITY study [17,18]. Real-world combined phaco-MIGS studies have further supported its effectiveness [19,20].

Despite this growing evidence base, data on standalone iStent infinite implantation in routine clinical practice remain limited. Therefore, the present study evaluates outcomes of standalone iStent infinite implantation in eyes with mild to severe POAG over 12 months, including subgroup analyses by preoperative IOP, glaucoma severity, medication burden, and lens status. To the best of our knowledge, this represents the first published real-world analysis of standalone iStent infinite outcomes in the literature.

## 2. Materials and Methods

### 2.1. Study Design and Participants

This was a retrospective case series of consecutive patients with POAG who underwent iStent infinite implantation as a standalone procedure between November 2022 and May 2024 by a single glaucoma surgeon at a private U.S. practice. Institutional Review Board exemption was obtained prior to chart review (Salus IRB, Number 25835, approval 9 October 2025); all patients had provided informed consent for their surgical procedures, and separate consent for retrospective inclusion of de-identified data was not required per the terms of the exemption. All procedures adhered to the tenets of the Declaration of Helsinki.

Inclusion criteria consisted of a diagnosis of POAG (mild, moderate or severe) and a need for additional glaucoma treatment due to above-goal IOP or medication burden, and/or poor topical medication adherence or tolerance. Glaucoma severity was categorized in accordance with the Centers for Medicare & Medicaid Services (CMS) staging criteria, using visual field parameters recorded at baseline. Patients were required to have a minimum of 12 months of follow-up data. Exclusion criteria included angle-closure glaucoma, active ocular inflammation, prior intraocular surgery within 3 months, or anatomical contraindications to trabecular micro-bypass implantation.

### 2.2. Device Description, Surgical Technique, and Postoperative Care

As detailed previously, the iStent infinite system (Glaukos Corporation, Aliso Viejo, CA, USA) contains three preloaded, titanium stents with lateral outlet lumens designed to bypass the trabecular meshwork into Schlemm’s canal [18]. Under gonioscopic guidance, the injector is used to place the stents across 4–6 clock hours of the nasal angle (each stent placed approximately two clock hours apart). Confirmation of proper stent position is then verified with gonioscopy. Intracameral moxifloxacin (generic formulation) was administered in all cases. Following the procedure, patients were prescribed a 10-day course of topical Bromfenac 0.07% (generic formulation) once daily. No washout of glaucoma medications was performed before surgery.

### 2.3. Outcome Measures

Primary effectiveness outcomes were the change in intraocular pressure (IOP) and topical medication burden at 12 months compared with baseline. Secondary effectiveness outcomes included the proportions of eyes achieving IOP thresholds of ≤18, ≤15, and ≤12 mmHg and the distribution of topical medication burden. Outcomes were evaluated for the overall cohort and for two preoperative IOP subgroups: eyes with a baseline of IOP ≤ 18 mmHg (goal medication-reduction group) and eyes with a baseline of IOP > 18 mmHg (goal IOP reduction group). Additional subgroup analyses of IOP outcomes were also performed according to glaucoma severity (mild, moderate, severe) and preoperative medications. Exploratory subgroup analyses were performed to evaluate outcomes according to lens status (phakic vs. pseudophakic). Safety outcomes included adverse events and additional glaucoma procedures.

Time-to-secondary-intervention analyses were performed using Kaplan–Meier analyses under three definitions of secondary intervention: any IOP-related intervention, incisional or laser procedures only, and incisional procedures only.

### 2.4. Statistical Analysis

Continuous outcomes, including mean IOP and mean medication count at each follow-up timepoint, were analyzed using linear mixed models with random subject intercepts to account for the repeated-measures nature of the data and the variability in visit attendance across timepoints. Medication count data and dichotomous IOP threshold outcomes were analyzed using generalized linear mixed models with random subject intercepts. This modeling approach utilizes all available data from all subjects at every time point where observations exist, without requiring complete case data or last-observation-carried-forward imputation, and does not make assumptions about the mechanism of missingness. Subgroup analyses were parameterized similarly to the primary analyses with the addition of a subgroup class variable for baseline IOP (≤18 mmHg vs. >18 mmHg), glaucoma severity (mild, moderate, or severe), and baseline medication burden (0–2 vs. ≥3 medications). All subgroup analyses were exploratory in nature and were not adjusted for multiple comparisons; findings from these analyses should therefore be interpreted as hypothesis-generating rather than confirmatory.

Two pre-specified sensitivity analyses were performed. Sensitivity Analysis 1 censored the 4 eyes that underwent secondary incisional glaucoma surgery at the time of their reintervention, to evaluate whether inclusion of post-reintervention data influenced primary outcomes. Sensitivity Analysis 2 excluded the 6 eyes with prior procedural pharmaceutical implantation or Ahmed tube shunt, to assess whether residual effects of these prior interventions may have confounded baseline or month 12 outcomes.

Data are presented as mean ± standard deviation (SD) or as count and percentage. A *p*-value < 0.05 was considered statistically significant. Statistical analysis was performed with Microsoft Excel version 16 (Microsoft Corporation, Redmond, WA, USA) and SAS version 9.4 M9 (SAS, Cary, NC, USA) statistical software. 

## 3. Results

### 3.1. Study Population

Fifty-one eyes of 51 patients underwent standalone iStent infinite implantation and were included in the analysis. The mean age was 66.5 ± 10.8 years, and 56.9% were male. All eyes had primary open-angle glaucoma, categorized as mild in 43.1%, moderate in 37.3%, and severe in 19.6%. Mean preoperative IOP was 20.1 ± 5.35 mmHg on 2.1 ± 1.24 medications. At baseline, 41.2% of eyes had IOP ≤ 18 mmHg, and 47.1% were receiving ≥ 3 medications. Prior ocular procedures were common (88%), including prior SLT in 74.5% of eyes, MIGS in 5.8% of eyes, Ahmed tube shunt in 3.9% of eyes, and bimatoprost implant in 7.8% of eyes (Table 1). No medication washout was performed.

Baseline characteristics of eyes requiring secondary glaucoma intervention (n = 10) compared with those not requiring reintervention (n = 41) are presented in Table 1. Among eyes requiring secondary intervention, mean baseline IOP was 23.2 ± 7.5 mmHg, with 70% having baseline IOP > 18 mmHg and none with baseline IOP < 15 mmHg. Fifty percent of eyes requiring secondary intervention were receiving ≥ 3 glaucoma medications at baseline. Glaucoma severity was mild in 60%, moderate in 20%, and severe in 20% of eyes in this subgroup. All 10 eyes had a history of prior glaucoma procedures. Compared with eyes not requiring reintervention—in whom the mean baseline IOP was 19.3 ± 4.5 mmHg and 46.3% were on ≥3 medications—eyes requiring secondary intervention tended to have higher baseline IOP and greater prior treatment burden, consistent with a more treatment-resistant disease profile in this subgroup. Both eyes with a history of prior Ahmed tube shunt implantation (n = 2, 100%) were among those requiring secondary glaucoma intervention, compared with none of the eyes without prior tube surgery, suggesting that a history of prior tube shunt may identify eyes at higher risk of reintervention following standalone iStent infinite implantation—though the small number of affected eyes precludes definitive conclusions.

### 3.2. Intraocular Pressure and Medication Use, Overall Cohort

**Figure 1:**
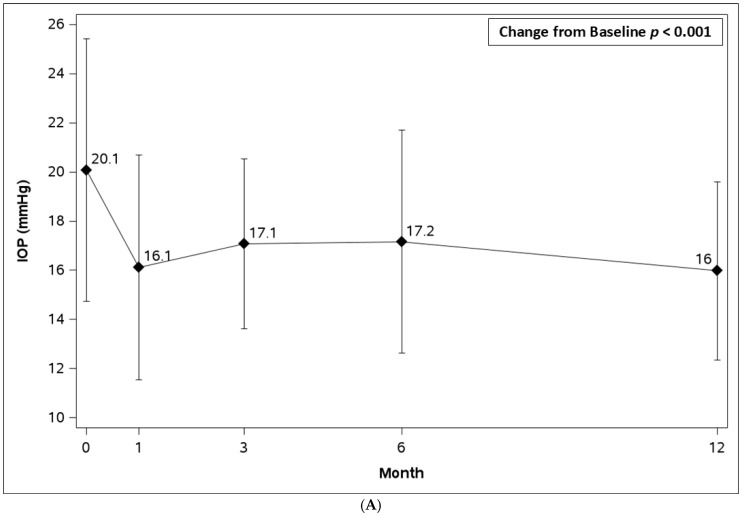

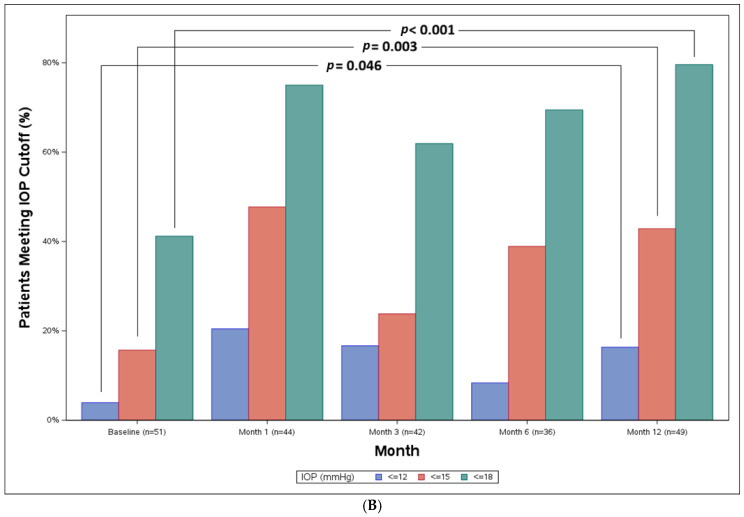
(**A**) Mean intraocular pressure (IOP) over 12 months following standalone iStent infinite implantation in the overall cohort (line graph). Vertical error bars indicate ± 1 standard deviation (SD). (**B**) Baseline vs. 12M proportions of eyes with IOP ≤ 18 mmHg, ≤15 mmHg, and ≤12 mmHg in the overall cohort (bar graph).

Mean IOP decreased from 20.1 ± 5.4 mmHg at baseline to 16.0 ± 3.6 mmHg at month 12 (n = 49), representing a mean reduction of 4.1 mmHg (−20.4%; *p* < 0.001). IOP reduction was observed at all follow-up time points, including month 1 (16.1 ± 4.6 mmHg), month 3 (17.1 ± 3.5 mmHg), month 6 (17.2 ± 4.5 mmHg), and month 12 (16.0 ± 3.6 mmHg) (Table 2, Figure 1A).

At month 12, 79.6% of eyes achieved IOP ≤ 18 mmHg compared with 41.2% at baseline, 42.9% achieved IOP ≤ 15 mmHg compared with 15.7% at baseline, and 16.3% achieved IOP ≤ 12 mmHg compared with 3.9% at baseline (all *p* < 0.05) (Figure 1B).

### 3.3. Medications Outcomes

Mean medication burden decreased from 2.1 ± 1.24 medications at baseline to 1.5 ± 1.2 at month 12, representing a 28.6% reduction (*p* < 0.001). Medication use decreased as early as month 1 (1.4 ± 1.3) and remained reduced through month 3 (1.6 ± 1.3), month 6 (1.5 ± 1.1), and month 12 (1.5 ± 1.2) (Table 2).

**Figure 2:**
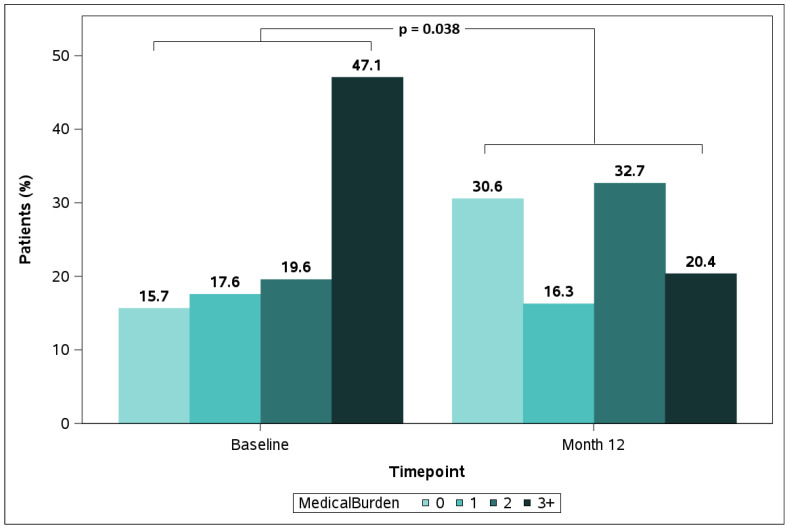
Medication burden distribution over time.

The proportion of medication-free eyes increased from 15.7% at baseline to 30.6% at month 12, while the proportion of eyes requiring ≥ 3 medications decreased from 47.1% to 20.4%. Overall, the distribution of medication burden shifted toward fewer agents by month 12 (Figure 2).

### 3.4. Subgroup Analysis by Preoperative IOP

Eyes with baseline IOP > 18 mmHg (n = 30, goal IOP reduction) demonstrated an IOP reduction from 23.0 ± 4.9 mmHg at baseline to 16.6 ± 3.9 mmHg at month 12, corresponding to a mean decrease of 6.4 ± 5.9 mmHg (−27.8% reduction, *p* < 0.001) (Table 3). This was accompanied by a non-significant reduction in mean medication burden (from 2.0 to 1.6 mean medications, *p* = 0.08) (Table 4).

Eyes with baseline IOP ≤ 18 mmHg (n = 21, goal medication reduction) experienced a significant reduction in medication burden, from 2.2 ± 1.1 mean medications at the baseline to 1.3 ± 1.3 at last follow-up (41% reduction, *p* < 0.001) (Table 4). This was accompanied by maintenance of stable IOP, with mean values of 15.9 ± 2.4 mmHg at baseline and 15.1 ± 3.2 mmHg at month 12 (*p* = 0.422) (Table 3).

### 3.5. Subgroup Analysis by Glaucoma Severity

IOP reductions were observed across all glaucoma severity levels. At month 12, mean IOP decreased from 21.6 ± 6.5 to 16.4 ± 4.3 mmHg in eyes with mild glaucoma (*p* < 0.001), from 18.4 ± 4.6 to 15.8 ± 3.1 mmHg in eyes with moderate glaucoma (*p* = 0.024), and from 19.9 ± 2.4 to 15.3 ± 2.9 mmHg in eyes with severe glaucoma (*p* = 0.008). Reductions were maintained across all severity groups throughout the follow-up (all *p* < 0.05) (Table 3).

Mean medications reduced from 1.9 to 1.3 in mild eyes (*p* = 0.028) and from 1.9 to 1.2 in moderate eyes (*p* = 0.004). In eyes with severe glaucoma, medication burden decreased from 2.7 to 2.4 medications; however, this reduction did not reach statistical significance (*p* = 0.377), likely reflecting the small sample size in this subgroup (n = 10) and the higher baseline medication burden in eyes with more advanced disease. Thus, there was no significant difference in the magnitude of IOP reduction across severity subgroups, while medication reduction outcomes were not uniform across severity levels and should be interpreted accordingly.

### 3.6. Subgroup Analysis by Preop Medications

In eyes receiving 0–2 medications at baseline, mean IOP decreased from 19.8 ± 3.8 mmHg preoperatively to 17.0 ± 2.9 mmHg at month 12 (14% reduction, *p* = 0.005). In eyes receiving ≥ 3 medications at baseline, mean IOP decreased from 20.4 ± 6.78 mmHg to 14.9 ± 4.03 mmHg at month 12 (27% reduction, *p* < 0.001; Table 3). There was no significant difference in the amount of IOP reduction experienced across subgroups based on preoperative medication burden. Mean medication use decreased from 1.1 to 0.8 (*p* = 0.19) in eyes receiving 0–2 medications at baseline, and from 3.2 to 2.3 in eyes receiving ≥ 3 medications at baseline (*p* < 0.001; Table 4).

### 3.7. Subgroup Analysis by Preop Lens Status

There were no statistically significant differences in IOP reduction or change in medication burden between phakic and pseudophakic eyes at 12 months (*p* ≥ 0.05 for all comparisons).

### 3.8. Safety Outcomes

No intraoperative adverse events occurred. Postoperatively, treatment-related adverse events included hyphema in two eyes (3.9%) on postoperative Day 1, which resolved spontaneously without sequelae at 1 week follow-up. Overall, secondary glaucoma intervention (incisional, laser, or procedural pharmaceutical) occurred in 10 eyes (19.6%). Incisional secondary glaucoma procedures were performed in four eyes (7.8%), including subconjunctival microshunt implantation in two eyes (ClearPath at months 5 and 12), trabeculotomy in one eye (month 9), and cyclophotocoagulation in one eye (month 12). Laser interventions with selective laser trabeculoplasty were performed in two eyes (3.9%) at months 4 and 12. Sustained-release pharmaceutical interventions were administered in four eyes (7.8%), including an intracameral bimatoprost implant in three eyes and an intracameral travoprost implant in one eye (all at month 6). No cases of persistent hypotony, device explantation, endophthalmitis, or other vision-threatening complications were observed.

Sensitivity Analysis 1—Censoring Eyes with Incisional Secondary Surgery

To evaluate whether inclusion of post-reintervention data in the primary analysis may have biased month 12 outcomes favorably, a sensitivity analysis was performed, censoring the four eyes that underwent secondary incisional glaucoma surgery at the time of their reintervention. In this censored analysis (n = 47 for IOP, n = 46 for medications at month 12), the mean IOP decreased from 20.1 ± 5.3 mmHg at baseline to 16.1 ± 3.5 mmHg at month 12 (−20.1%, *p* < 0.001), and the mean medication burden decreased from 2.1 ± 1.2 to 1.5 ± 1.2 (*p* < 0.001). These results are consistent with the primary analysis, confirming that inclusion of post-reintervention data did not materially influence the observed IOP or medication outcomes.

Sensitivity Analysis 2—Excluding Eyes with Prior Procedural Pharmaceutical Implant or Tube Implantation

To assess potential confounding from residual effects of prior procedural pharmaceutical implantation or Ahmed tube shunt, a sensitivity analysis was performed, excluding the six affected eyes (n = 45 at baseline). The mean time interval between prior procedural pharmaceutical or tube implantation and iStent infinite surgery in these eyes was 530 days (SD 387.8 days; range 107–1205 days). Following exclusion of these eyes, mean IOP decreased from 19.4 ± 4.8 mmHg at baseline to 15.4 ± 3.3 mmHg at month 12 (n = 44; −20.6%, *p* < 0.001), and mean medication burden decreased from 2.1 ± 1.2 at baseline to 1.6 ± 1.2 at month 12 (n = 42; *p* < 0.001). These findings are consistent with the primary analysis, confirming that the primary outcomes were not materially influenced by the inclusion of eyes with prior procedural pharmaceutical implantation or tube shunt surgery.

Time-to-Secondary-Intervention Analysis

**Figure 3:**
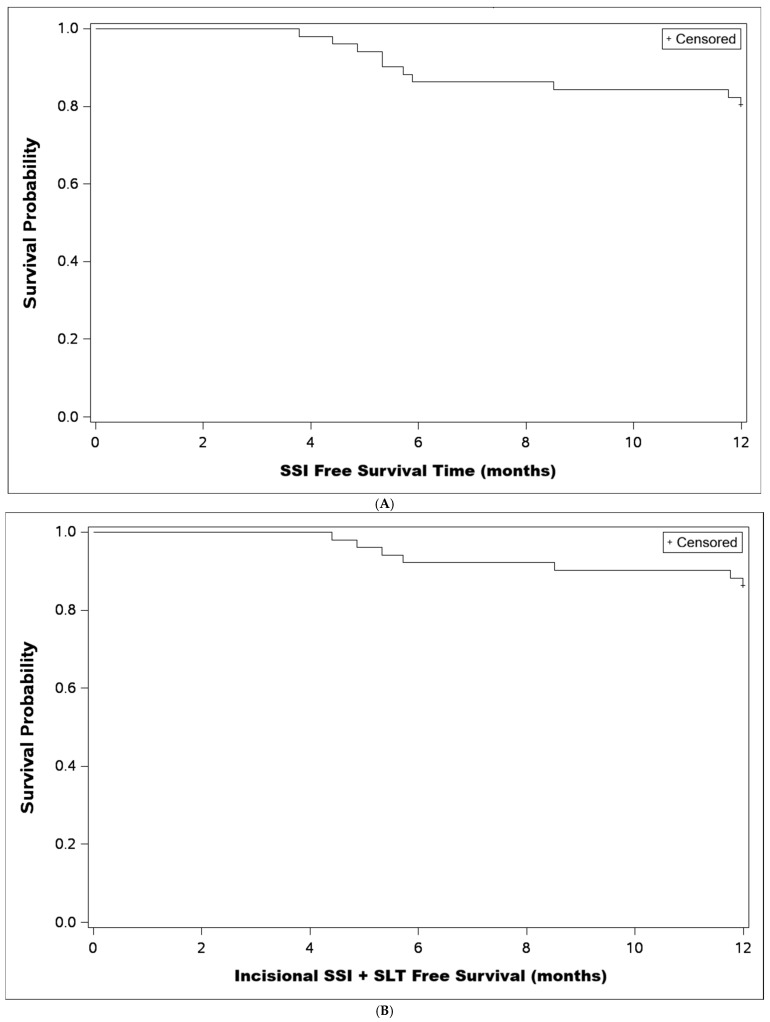

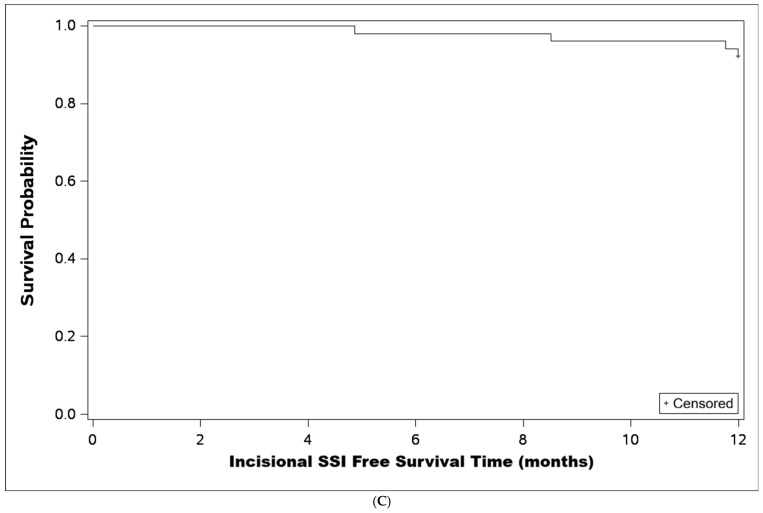
**Kaplan–Meier time-to-secondary-intervention survival curves.** (**A**) Freedom from any IOP-related secondary intervention (n = 10 events); estimated 12-month survival probability 80.4%. Tick marks indicate censored observations. SSI = secondary surgical intervention. (**B**) Freedom from incisional or laser secondary intervention (n = 6 events); estimated 12-month survival probability 86.3%. Tick marks indicate censored observations. SSI = secondary surgical intervention; SLT = selective laser trabeculoplasty. (**C**) Freedom from incisional secondary intervention only (n = 4 events); estimated 12-month survival probability 92.2%. Tick marks indicate censored observations. SSI = secondary surgical intervention.

Kaplan–Meier time-to-secondary-intervention analyses were performed under three definitions of secondary intervention. When defined as any IOP-related intervention (n = 10 total events), the estimated probability of remaining free from secondary intervention at 12 months was approximately 80.4% (Figure 3A). When restricted to incisional or laser procedures (n = 6 events), the 12-month freedom from intervention probability was approximately 86.3% (Figure 3B). When restricted to incisional procedures only (n = 4 events), the 12-month freedom from incisional reintervention probability was approximately 92.2% (Figure 3C). Across all three definitions, the majority of secondary interventions occurred between months 4 and 9, with survival probability remaining stable thereafter through month 12.

## 4. Discussion

This retrospective study evaluated the outcomes of standalone implantation of the iStent infinite trabecular micro-bypass in eyes with mild to severe open-angle glaucoma. The inclusion of consecutive cases treated in routine clinical practice in the United States enhances the external validity of the findings. As such, the results may more closely reflect real-world clinical performance compared with those derived from the more controlled conditions and restrictive inclusion criteria typical of prospective clinical trials.

At 12 months, standalone iStent infinite implantation demonstrated meaningful reductions in both intraocular pressure (IOP) and medication burden. Mean IOP decreased from 20.1 ± 5.4 mmHg at baseline to 16.0 ± 3.6 mmHg, corresponding to a reduction of 20.4%, while concurrently the mean number of medications decreased by 28.6%, from 2.1 ± 1.24 to 1.5 ± 1.2. These findings are consistent with prior real-world studies of iStent infinite. For example, Vest et al. reported a 23.8% reduction in IOP and a 23.2% decrease in medication burden at 12 months following combined iStent infinite implantation with cataract surgery in patients with mild-to-moderate primary open-angle glaucoma, supporting the effectiveness of the device across varying clinical settings [19].

The magnitude of IOP reduction observed with standalone iStent implantation has varied across different studies. A meta-analysis by Chen et al. [21] reported a mean IOP reduction of −2.64 mmHg and medication reduction of −1.71 at 6–18 months, while a more recent analysis by Tan et al. [22] demonstrated a broader range of IOP reduction (5.2–40.7%) and a mean decrease of approximately −4.9 mmHg at 24 months. These studies primarily evaluated earlier-generation iStent devices with the implantation of one or two stents, whereas the iStent infinite system utilizes three stents, which may enhance access to multiple collector channels and improve aqueous outflow. The variability in outcomes across studies may also be explained by underlying physiological factors. The effectiveness of trabecular bypass procedures depends on the patency and distribution of distal collector channels, which may be segmentally dysfunctional or obstructed in glaucoma. In addition, the proximity of the implant device to functional collector channels and the presence of episcleral venous pressure as a limiting factor may further influence the achievable IOP reduction [22]. Together, these factors likely contribute to the heterogeneity of reported outcomes and provide context for the magnitude of IOP reduction observed in the present study.

Reducing topical medication burden offers several important advantages. Glaucoma drops are associated with ocular surface toxicity, local and systemic adverse effects, and may increase the risk of surgical failure while negatively impacting quality of life. Adherence to topical therapy is often poor and declines further with increasing medication burden, thereby limiting treatment efficacy and contributing to disease progression. Moreover, even with proper use, topical medications are associated with intraocular pressure fluctuations, which may go undetected and further accelerate glaucoma progression [19,23].

In addition to mean reductions, clinically meaningful improvements were observed in IOP target attainment. The proportion of eyes achieving IOP thresholds of ≤18, ≤15, and ≤12 mmHg increased substantially at month 12, with an approximate two-fold, three-fold, and greater than four-fold increase, respectively. Similarly, the proportion of medication-free eyes doubled from 15.7% at baseline to 30.6% at month 12, while the proportion of eyes requiring ≥ 3 medications decreased by more than two-fold. These findings are consistent with those reported by Shultz et al., who demonstrated marked increases in the proportion of eyes achieving lower IOP thresholds and reductions in medication burden following iStent infinite implantation with phacoemulsification [20].

Subgroup analyses further highlight the differential response based on baseline IOP. Eyes with baseline IOP > 18 mmHg demonstrated a greater relative IOP reduction (27.8%) and only marginal medication reduction (20%), whereas eyes with baseline IOP ≤ 18 mmHg maintained stable IOP with a concomitant significant reduction (41%) in medication burden. This observation is consistent with the well-established principle that eyes with higher preoperative IOP tend to exhibit greater absolute and relative reductions following glaucoma procedures, as reported across multiple studies. From a physiological standpoint, these devices enhance aqueous outflow through Schlemm’s canal and distal collector channels, and the magnitude of IOP reduction is partly driven by the pre-existing pressure gradient across the trabecular meshwork. Accordingly, higher baseline IOP provides greater potential for reduction, whereas eyes with lower baseline IOP are more likely to benefit from medication reduction while maintaining target pressure, a phenomenon similarly described in pivotal trabecular bypass trials such as those by Samuelson et al. [24].

IOP reductions were observed across all glaucoma severity levels, with statistically significant decreases in IOP maintained through 12 months in mild, moderate and severe subgroups. Interestingly, the greatest reductions were observed in both mild and severe glaucoma groups, whereas the moderate group demonstrated comparatively smaller reductions. This pattern appears to be primarily driven by differences in baseline IOP within each subgroup, as eyes classified as mild and severe glaucoma in this cohort had relatively higher baseline IOP compared with the moderate group, reinforcing the concept that baseline IOP, rather than disease severity alone, is a key determinant of postoperative IOP reduction following trabecular bypass interventions, consistent with broader observations in the MIGS literature [25].

However, outcomes were not uniform across severity subgroups when medication reduction was considered. Medication reduction in eyes with severe glaucoma did not reach statistical significance (*p* = 0.377), likely reflecting limited statistical power in this small subgroup (n = 10). Furthermore, a mean month 12 IOP of 15.3 mmHg on 2.4 medications in severe eyes, while representing a meaningful reduction from baseline, may not achieve the lower target IOP thresholds typically recommended for advanced glaucoma management. Ultimately, astute clinical judgment and individualized treatment targets are needed to guide what intervention(s) these patients receive.

A similar pattern was observed when stratifying by preoperative medication burden. Eyes receiving ≥ 3 medications at baseline demonstrated greater IOP reduction (27%) compared with those on 0–2 medications (14%), consistent with a higher-IOP, more treatment-resistant disease profile in this subgroup. An exploratory analysis by lens status revealed no significant differences in IOP or medication outcomes between phakic and pseudophakic eyes (*p* ≥ 0.05), suggesting treatment efficacy is largely independent of lens status in this context, though confirmation in larger studies is warranted.

The robustness of the primary findings was supported by two pre-specified sensitivity analyses. Censoring the four eyes that underwent incisional secondary surgery at the time of reintervention yielded consistent month 12 results (mean IOP 16.1 ± 3.5 mmHg, *p* < 0.001; mean medications 1.5 ± 1.2, *p* < 0.001), confirming that inclusion of post-reintervention data did not materially bias primary outcomes. Excluding the six eyes with prior procedural pharmaceutical implantation or Ahmed tube surgery similarly demonstrated consistent findings (mean IOP 15.4 ± 3.3 mmHg, *p* < 0.001; mean medications 1.6 ± 1.2, *p* < 0.001), with a mean interval of 530 days between prior procedure and iStent infinite implantation in these eyes.

Kaplan–Meier analysis demonstrated 12-month freedom from any secondary intervention of approximately 80.4%, rising to 86.3% when restricted to incisional or laser procedures, and 92.2% when restricted to incisional procedures only—with the majority of interventions occurring between months 4 and 9.

Standalone iStent infinite implantation demonstrated a favorable safety profile, with no intraoperative complications and only transient postoperative events. Hyphema occurred in 3.9% of eyes and resolved without sequelae. No cases of persistent hypotony, endophthalmitis, device explantation, or other vision-threatening complications were observed, consistent with prior reports of trabecular micro-bypass procedures.

The overall rate of secondary glaucoma interventions was 19.6%; however, this included incisional, laser, and procedural pharmaceutical interventions. Incisional secondary surgical interventions occurred in four eyes (7.8%), while the remaining interventions consisted of selective laser trabeculoplasty (3.9%) and sustained-release pharmacologic implants (7.8%). The distinction between SSI types is clinically relevant, given the different risk profiles of various interventions.

When considering incisional interventions alone, the observed rate is higher than that reported in the pivotal study by Sarkisian et al. (4.2%) and the INTEGRITY study (~1.1%), likely reflecting the more treatment-resistant nature of this real-world cohort, lower thresholds for escalation in routine practice, and differences in reporting definitions across studies [17,18].

This study has several limitations. Its retrospective single-center design and absence of a control group limit causal inference, and the conclusions should be interpreted as suggesting potential benefit requiring confirmation in larger prospective controlled studies. The absence of functional and structural outcome measures represents an important limitation of the present study. Endpoints, including best-corrected visual acuity, visual field stability or progression, optic nerve head parameters, retinal nerve fiber layer thickness on optical coherence tomography, and endothelial cell density, were not systematically captured in this retrospective dataset. Without these data, it is not possible to determine whether the observed reductions in IOP and medication burden translated into meaningful preservation of visual function or structural stability over the 12-month follow-up period. Future prospective studies should incorporate these endpoints to provide a more comprehensive assessment of the clinical impact of standalone iStent infinite implantation. No preoperative medication washout was performed, consistent with real-world practice, but baseline IOP values may underestimate the true pressure-lowering capacity of the device. The dataset was identified through an EHR query requiring 12-month follow-up availability, introducing the possibility of attrition bias. The demographic composition—72.5% Black/African American—reflects local practice demographics and represents a strength for representation of a high-risk population, but may limit generalizability to other settings. No exploratory subgroup analysis by race was performed in the current study, and whether outcomes differed across racial groups remains an area warranting investigation in future larger-scale studies.

The strengths of this study include the inclusion of consecutive real-world cases across a broad spectrum of glaucoma severity, providing clinically relevant insight into the performance of standalone iStent infinite implantation in a population at high glaucoma risk.

## 5. Conclusions

Standalone iStent infinite implantation resulted in significant reductions in intraocular pressure and medication burden over 12 months, with a favorable safety profile and no vision-threatening complications. Outcomes aligned with preoperative treatment goals, with greater IOP reduction in eyes with higher baseline IOP and greater medication reduction in eyes with lower baseline IOP. These findings suggest potential benefit across a range of glaucoma severities and preoperative treatment profiles, and support further investigation of the standalone iStent infinite in larger prospective controlled studies.

## Figures and Tables

**Table 1:** Baseline patient and eye characteristics. Data presented as n (%) or mean ± SD.

	All Eyes (n = 51)	SSI (n = 10)	No SSI (n = 41)
**Age (years)**	66.5 ± 10.8	64.1 ± 9.7	67.1 ± 11.1
**Gender**			
Male	29 (56.9%)	5 (50.0%)	24 (58.5%)
Female	22 (43.1%)	5 (50.0%)	17 (41.5%)
**Race**			
Asian	1 (2.0%)	0 (0.0%)	1 (2.4%)
Black/African American	37 (72.5%)	7 (70.0%)	30 (73.2%)
Hispanic	1 (2.0%)	1 (10.0%)	0 (0.0%)
White	12 (23.5%)	2 (20.0%)	10 (24.4%)
**Laterality**			
Right	26 (51.0%)	5 (50.0%)	21 (51.2%)
Left	25 (49.0%)	5 (50.0%)	20 (48.8%)
**Preoperative IOP (mmHg)**	20.1 ± 5.35	23.2 ± 7.5	19.3 ± 4.5
**Medications (#)**	2.1 ± 1.24	2.2 ± 1.0	2.0 ± 1.3
**Baseline Medication Category**			
0	8 (15.7%)	1 (10.0%)	7 (17.1%)
1	9 (17.6%)	1 (10.0%)	8 (19.5%)
2	10 (19.6%)	3 (30.0%)	7 (17.1%)
≥3	24 (47.1%)	5 (50.0%)	19 (46.3%)
**Pachymetry (μm)**	546.4 ± 41.7 (n = 50)	567.1 ± 15.4 (n = 9)	541.8 ± 44.3
**Glaucoma Severity**			
Mild	22 (43.1%)	6 (60.0%)	16 (39.0%)
Moderate	19 (37.3%)	2 (20.0%)	17 (41.5%)
Severe	10 (19.6%)	2 (20.0%)	8 (19.5%)
**Lens Status**			
Phakic	21 (41.2%)	2 (20.0%)	19 (46.3%)
Pseudophakic	30 (58.8%)	8 (80.0%)	22 (53.7%)
**Prior MIGS** *	3 (5.8%)	1 (10.0%)	2 (4.9%)
**Prior SLT**	38 (74.5%)	6 (60.0%)	32 (78.0%)
**Prior Durysta (bimatoprost implant)**	4 (7.8%)	0 (0.0%)	4 (9.8%)
**Prior Ahmed Tube**	2 (3.9%)	2 (20.0%)	0 (0.0%)

IOP = intraocular pressure, MIGS = minimally invasive glaucoma surgery, SSI = secondary glaucoma intervention, SLT = selective laser trabeculoplasty. * Prior MIGS includes iStent, OMNI, Hydrus.

**Table 2:** IOP and medication outcomes of all eyes at all timepoints. Data presented as mean ± SD.

Timepoint	N	Mean IOP (mmHg)	Mean Medications (n)
Baseline	51	20.1 ± 5.35	2.1 ± 1.24
Month 1	44	16.1 ± 4.57	1.4 ± 1.3
Month 3	42	17.1 ± 3.46	1.6 ± 1.3
Month 6	36	17.2 ± 4.53	1.5 ± 1.1
Month 12	49	16.0 ± 3.63	1.5 ± 1.2

IOP = intraocular pressure.

**Table 3:** IOP outcomes of eyes at the baseline and 12 months follow up split by group. Data presented as mean ± SD.

Subgroup	Baseline IOP (mmHg)	Month 12 IOP (mmHg)	Change	*p* Value
**Baseline IOP subgroup**				
≤18 mmHg	15.9 ± 2.39 (n = 21)	15.1 ± 3.18 (n = 21)	−0.81 ± 3.36	0.422
>18 mmHg	23.0 ± 4.89 (n = 30)	16.6 ± 3.85 (n = 28)	−6.4 ± 5.87	<0.001 *
**Glaucoma severity subgroup**				
Mild	21.6 ± 6.51 (n = 22)	16.4 ± 4.31 (n = 22)	−5.2 ± 6.66	<0.001 *
Moderate	18.4 ± 4.60 (n = 19)	15.8 ± 3.12 (n = 18)	−2.6 ± 4.87	0.024 *
Severe	19.9 ± 2.38 (n = 10)	15.3 ± 2.92 (n = 9)	−4.6 ± 4.00	0.008 *
**Baseline medications subgroup**				
0–2 Medications	19.8 ± 3.76 (n = 27)	17.0 ± 2.89 (n = 25)	−2.8 ± 4.31	0.005 *
≥3 Medications	20.4 ± 6.78 (n = 24)	14.9 ± 4.03 (n = 24)	−5.5 ± 6.54	<0.001 *

IOP = intraocular pressure. * Statistically significant.

**Table 4:** Medication burden of eyes at baseline and 12 months follow-up split by group. Data presented as mean ± SD.

Subgroup	Baseline Medications (n)	Month 12 Medications (n)	Mean Change (n)	*p* Value
**Baseline IOP subgroup**				
≤18 mmHg	2.2 ± 1.1	1.3 ± 1.3	−0.9	<0.001 *
>18 mmHg	2.0 ± 1.3	1.6 ± 1.2	−0.4	0.08
**Glaucoma severity subgroup**				
Mild	1.9 ± 1.3	1.3 ± 1.0	−0.6	0.028 *
Moderate	1.9 ± 1.1	1.2 ± 1.2	−0.7	0.004 *
Severe	2.7 ± 1.3	2.4 ± 1.2	−0.3	0.377
**Baseline medications subgroup**				
0–2 Medications	1.1 ± 0.8	0.8 ± 0.9	−0.3	0.19
≥3 Medications	3.2 ± 0.4	2.3 ± 1.0	−0.9	<0.001 *

IOP = intraocular pressure. * Statistically significant.

## Data Availability

Data are available upon reasonable request from the corresponding author.
